# Effect of small interfering RNA 3'-end overhangs on chemosensitivity to thymidylate synthase inhibitors

**DOI:** 10.1186/1758-907X-2-1

**Published:** 2011-01-19

**Authors:** John C Schmitz, Edward Chu

**Affiliations:** 1 VACT Healthcare System, VACT Cancer Center, West Haven, CT, USA; 2 Department of Medicine and Pharmacology, Developmental Therapeutics Program, Yale Cancer Center, Yale University School of Medicine, New Haven, CT, USA

## Abstract

**Background:**

Small interfering RNAs (siRNAs) are double-stranded RNAs that effectively inhibit expression of its complimentary target mRNA. Standard siRNAs contain two nucleotide overhangs on their 3' end. While these overhangs are usually comprised of deoxythymidines (dT), it has been shown that any nucleotide can be used on the 3' end without affecting RNAi silencing.

**Results:**

It was recently shown that extension of the 3' end to five or eight dT molecules allows siRNAs to be effectively complexed with linear polyethylenimine (PEI), leading to enhanced cellular uptake and intracellular release. Here, we provide further evidence that only extended or 'sticky' siRNAs complexed with PEI result in significant target knockdown. However, when investigating the potential effects of these extended siRNAs on growth of human colon cancer RKO cells, we observed a dose-dependent reversal of cytotoxicity of a thymidylate synthase-targeted siRNA. In contrast, siRNAs with uridine overhangs maintained their growth inhibitory effects. We further demonstrated that dT-containing siRNAs prevented the cytotoxic effects of thymidylate synthase (TS) inhibitor compounds, such as ZD1694 and 5'-fluoro-deoxyuridine, while having no deleterious effect on cisplatin toxicity. We show that this rescue effect results from the rapid degradation of the siRNA.

**Conclusions:**

Given that TS is an important enzyme for cell growth and proliferation and that its expression is controlled by multiple pathways, the rescue of its growth inhibitory effects may have unintended consequences. As siRNAs are being developed as therapeutic molecules, it will be important to avoid such off-target effects due to dT release. Hence, siRNAs should contain only uridine residues in their 3'-end overhangs.

## Background

Thymidylate synthase (TS) is a folate-dependent enzyme that catalyzes the reductive methylation of deoxyuridine monophosphate (dUMP) to deoxythymidine monophosphate (dTMP) [1]. Once synthesized, dTMP is subsequently metabolized intracellularly to deoxythymidine triphosphate (dTTP), a key nucleotide for DNA replication and repair. Although dTMP can be formed via the salvage pathway, a reaction catalyzed by thymidine kinase, the TS-catalyzed reaction provides the only intracellular *de novo *source of dTMP. As such, inhibition of this enzymatic step results in suppression of cellular growth and proliferation. Given the central role that TS plays in cellular proliferation, TS has been an important target for cancer chemotherapy for over 40 years [2,3].

Previous studies from our lab identified a small interfering RNA (siRNA) directed against the 3'-untranslated region (UTR) of human TS mRNA that was able to potently and specifically inhibit TS expression [4]. This siRNA exhibited a high level of specificity for TS mRNA as we were unable to identify off-target effects. In addition, this molecule effectively prevented the induction of TS protein following exposure to TS inhibitor compounds, such as the fluoropyrimidine 5-fluorodeoxyuridine and various antifolate analogs. Furthermore, treatment with this siRNA restored chemosensitivity to resistant human colon cancer RKO-HTStet cells that overexpressed TS by 15-fold. This work provided new insights towards the development of siRNAs as potential novel therapeutic molecules.

Two major issues confronting the development of siRNA therapeutics are their specificity and efficiency of delivery into target cells. Significant efforts have been placed, therefore, on developing nanoparticle technologies to facilitate siRNA cellular uptake. A wide range of molecules have been developed as delivery systems, and they include cationic lipids, carbon nanotubes, poly(lactic-coglycolic) acid (PLGA), polyethylenimine (PEI), peptides, dendrimers, and silicon and gold microparticles [5-11]. PEI has been used as an effective DNA plasmid delivery vehicle but has limited capacity for siRNA delivery [5,12]. Recently, Bolcato-Bellemin and colleagues extended the 3' end of the siRNA creating longer complimentary overhangs of five or eight nucleotides, thereby allowing PEI to effectively deliver these siRNA molecules into cells [13]. They hypothesized that the 'sticky' siRNAs or sticky end siRNAs (ssiRNAs) form gene-like concatemers with greater electrostatic interaction with PEI, thereby resulting in less cell surface polyanion exchange, enhanced cellular uptake, and eventually greater intracellular release of siRNA. Herein, we provide further evidence for the ability of PEI to efficiently deliver extended siRNAs, but not standard siRNAs, into human colon cancer RKO cells. As we further investigated the effects of the 3' overhangs on cell growth, we observed that the extended TS-targeted ssiRNA displayed a reduced level of cytotoxicity. Our findings show that as the siRNA is degraded, the deoxythymidine (dT) nucleotides on the 3' end are released, which then rescues cells from the cytotoxic effects of the TS-targeted siRNA. Additionally, we demonstrate that this release of dTMP from siRNA degradation is able to rescue cells from the cytotoxic effects of TS inhibitor compounds. The potential implication of these findings on the therapeutic efficacy of TS-associated cancer chemotherapy is discussed.

## Results

**Figure 1:**
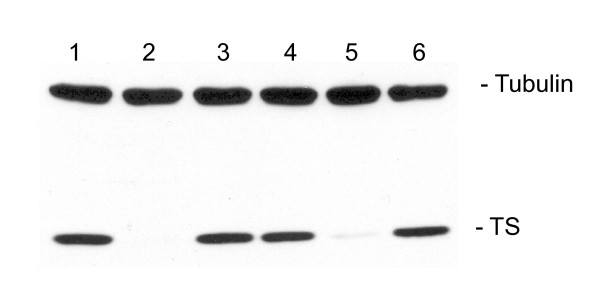
**Comparison of Lipofectamine 2000 (LF2000) and polyethylenimine (PEI) for siRNA transfection**. RKO cells were transfected with TS6 siRNAs (100 nM) complexed either LF2000 (lanes 1-3) or linear jetPEI (lanes 4-6). After 48 h, cells were harvested and processed for western blot analysis as described in the Methods section. Lane 1 contains cell lysates from LF2000-treated cells; lane 2, TS6 siRNA; lane 3, TS6 mismatch siRNA; lane 4, TS6 siRNA; lane 5, TS6 sticky end siRNA (ssiRNA); lane 6, TS6 mismatch ssiRNA.

The use of siRNAs to target and suppress specific genes has been a significant advance in studies of gene expression and function. However, the potential role of siRNAs as therapeutic molecules has been limited, in large part due to issues relating to stability, cellular uptake, and specificity of delivery into target tissues. Significant efforts have focused on developing delivery systems that encapsulate the siRNA in nanoparticles and then target the nanoparticle with small peptides, molecules, and/or antibodies against tissue-specific and/or tumor-specific receptors. Commercially available cationic lipids have been available for many years and represent ideal tools for cell culture delivery of nucleic acids. As seen in Figure 1, Lipofectamine 2000 (LF2000) was able to deliver the TS6 TS-targeted siRNA into human RKO colon cancer cells resulting in > 95% knockdown of the target protein (Figure 1, lane 2). While LF2000 has been widely used for *in vitro *tissue culture experiments, these lipid carriers have not been used for *in vivo *studies as they are associated with alteration of gene expression profiles, increased host toxicities, and activation of host immune response [14-17]. A different cationic molecule, PEI, has been used for *in vitro *and *in vivo *delivery of both DNA plasmids and siRNA [8,12]. However, we were unable to knockdown TS expression with linear PEI in combination with TS6 siRNA (Figure 1, lane 4). Bolcato-Bellemin *et al*. recently showed that PEI can deliver siRNA when the siRNA 3'-end overhangs, normally two deoxythymidine nucleotides in length, are extended to either five or eight nucleotides [13]. The overhangs on either end of the siRNA are made complimentary to allow for the formation of long gene-like concatemers that allow them to be effectively complexed by PEI and then released once inside the cell. This extended siRNA is termed 'sticky' siRNA or ssiRNA. When the TS6 siRNA was modified with 5 dT on the sense strand and 5 deoxyadenosine (dA) on the antisense strand to form TS6 ssiRNA, PEI was able to efficiently deliver and release TS6 ssiRNA into human colon cancer RKO cells, resulting in enhanced knockdown of TS protein levels (Figure 1, lane 5). As an important control, the modified mismatch control ssiRNA complexed with PEI had absolutely no effect on expression of TS protein (Figure 1, lane 6).

**Figure 2:**
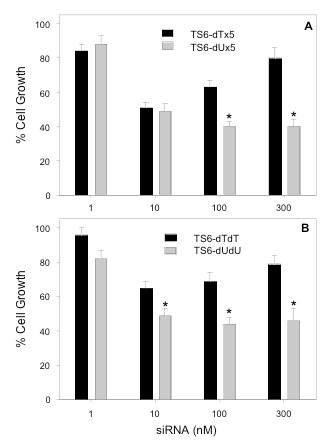
**Effect of ssiRNA/siRNA transfection on RKO cell growth**. **(a) **RKO cells were transfected with TS6 ssiRNA containing dTx5 (black bars) or dUx5 overhangs (gray bars) at the indicated concentrations. **(b) **RKO cells were transfected with TS6 siRNA containing dTdT (black bars) or dUdU overhangs (gray bars) at the indicated concentrations. After 96 h, a WST-1 cell proliferation assay was performed. Mock-transfected cells (LF2000 alone) were normalized to 100%. Values represent the mean ± standard error of the mean (SEM) from at least four separate experiments performed in duplicate. *P values < 0.01 versus dT-containing ssiRNAs or siRNAs at the same concentration.

To determine the effect of these extensions at the 3'-end of the siRNA on the ability of the siRNA to inhibit cell growth, we performed a series of cell proliferation experiments. At a concentration of 10 nM, TS6 ssiRNA suppressed cell growth by 50% (Figure 2a). However, at higher ssiRNA concentrations, the growth inhibitory effect was actually reversed. At the highest concentration of ssiRNA tested (300 nM), cell growth was minimally suppressed compared with untreated cells. We then synthesized an ssiRNA containing deoxyuridine (dU) instead of dT overhangs on the sense strand for comparison with the dT/dA ssiRNA (the antisense strand still contained 5 dA overhangs). As seen in Figure 2a, treatment with the dU-containing ssiRNA yielded a similar level of suppression of cell growth at 10 nM as the dT-ssiRNA. However, at higher concentrations, the growth inhibitory effects were not reversed as observed with treatment with the dT-ssiRNA. One possible explanation is that the dT-containing sense strand, upon release from the RISC, was being degraded intracellularly, resulting in the release of dTMP. This nucleotide is eventually metabolized within the cell to the dTTP triphosphate metabolite, which is an essential precursor for DNA biosynthesis and DNA repair. Once formed, dTTP would be able to rescue against the cytotoxic effects of the TS6 siRNA. We next determined whether this effect was, in fact, being observed with siRNAs containing standard dTdT overhangs. As seen in Figure 2b, TS6 siRNA has significantly less inhibitory effect on growth of RKO cells at all concentrations when compared to the siRNA with dU overhangs. Of note, addition of 10 μM thymidine to the cell culture medium completely reversed the growth inhibitory effects after 10 nM ssiRNA/siRNA transfection (96% ± 5 vs LF2000-treated cells). This protective effect has been previously reported by us and others using the same siRNA sequence [4,18].

**Figure 3:**
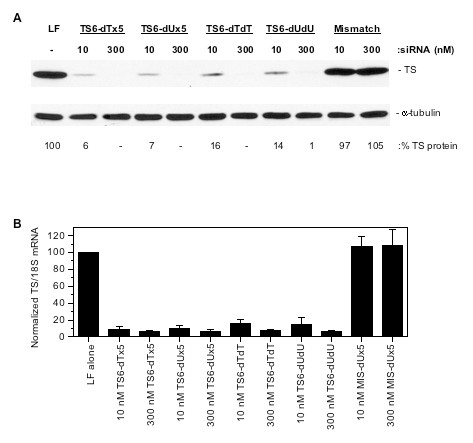
**Effect of ssiRNA/siRNA transfection on TS protein and mRNA expression**. RKO cells were transfected with TS6 siRNAs using LF2000. After 48 h, cells were harvested for western blot **(a) **or qPCR analysis **(b) **as described in the Methods section. (a) Representative western blot from three similar experiments. The percentage TS protein knockdown was quantified after scanning and analysis by ImageJ software. (b) Relative mRNA levels after siRNA transfection. Values represent the mean ± standard deviation (SD) from three individual experiments analyzed in triplicate. TS/18S values were normalized to the value of cells treated with LF2000 alone, which was set to 100.

To confirm that TS protein and TS mRNA levels were decreased after siRNA transfection, western blot and quantitative (q)PCR analyses were performed. As shown in Figure 3a, transfection of 10 nM of either TS6 ssiRNA resulted in > 90% decrease in TS protein. Upon 300 nM transfection, TS protein was undetectable. Similar observations were seen after standard TS6 siRNAs resulting in > 84% knockdown of TS protein after 10 nM transfection. Analysis of the relative levels of TS mRNA after transfection with the various siRNAs essentially mirrored the results observed with western blot analysis (Figure 3b).

**Figure 4:**
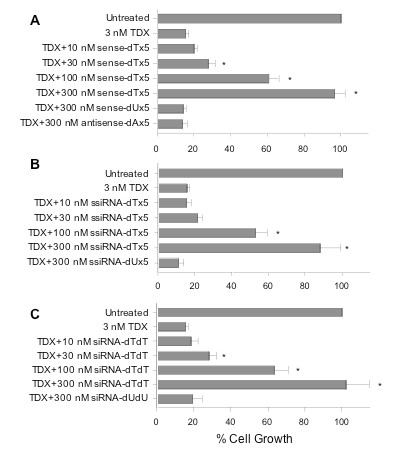
**Effect of RNA/ssiRNA/siRNA transfection on ZD1694 toxicity**. RKO cells were exposed to 3 nM ZD1694 (TDX) with or without **(a) **RNA, **(b) **ssiRNA, or **(c) **siRNA transfection. After 96 h, cell growth was determined by the WST-1 assay. Untreated cells (LF2000 alone) were normalized to 100%. Values represent the mean ± standard error of the mean (SEM) from at least four separate experiments performed in duplicate. *P values < 0.05 versus ZD1694 treatment alone.

To support the hypothesis that dTMP release was potentially rescuing the cytotoxicity caused by siRNA-mediated TS inhibition, we performed a series of growth experiments combining dT-ssiRNA with the TS inhibitor ZD1694 (also known as TDX). This antifolate compound is a specific inhibitor of TS, and its inhibitory effects on cell growth are completely reversed with the addition of exogenous thymidine in the cell culture medium [19,20]. Exposure of RKO cells to 3 nM ZD1694 resulted in 85% growth suppression (Figure 4a). When the sense strand alone (containing 5xdT 3'-end overhang) was transfected simultaneously with 3 nM ZD1694, a dose-dependent rescue from the ZD1694 cytotoxic effects was observed. RNA concentrations as low as 30 nM demonstrated significant enhancement of cell growth. At the highest concentration used in this experiment (300 nM), the growth inhibitory effects of ZD1694 were completely reversed. As an important control, 300 nM of the sense strand containing the 5xdU overhang was unable to rescue the inhibitory effects of ZD1694. Similarly, no rescue effect was observed when RKO cells were treated with the antisense strand containing the 5xdA overhang.

While a single-stranded RNA is most likely degraded rapidly to individual nucleotides, it was not clear as to whether the double-stranded ssiRNA, once transfected and incorporated into the RNA-induced silencing complex (RISC), would degrade rapidly enough to release sufficiently high levels of dTMP. To determine the effect of transfection of the double-stranded ssiRNA on ZD1694 cytotoxicity, the TS6 mismatch ssiRNA was transfected into RKO cells. Since TS6 ssiRNA has effects on cell growth, we chose the mismatch ssiRNA as it had absolutely no effects on growth of RKO cells on its own. As observed with the single-stranded dT-containing RNA, the TS6 mismatch ssiRNA was also able to reverse the inhibitory effects of ZD1694. In contrast, a TS6 mismatch ssiRNA with 5xdU overhangs was unable to reverse ZD1694 cytotoxicity (Figure 4b).

To determine if standard siRNAs would have the same impact on the growth inhibitory effects of ZD1694 as extended ssiRNAs, a series of experiments were conducted with the mismatch siRNA. As seen in Figure 4c, siRNAs with dTdT overhangs were able to reverse ZD1694 toxicity in a dose-dependent fashion. These results were not significantly different from those utilizing ssiRNA (Figure 4b). Since siRNAs contain four dT molecules per siRNA and the ssiRNA contain five dT molecules per ssiRNA, it is not surprising that their ability to rescue from ZD1694 toxicity is virtually identical. Two additional dTdT-containing siRNAs targeting other mRNAs (firefly luciferase and human epidermal growth factor receptor 2 (HER2)) were also able to reverse ZD1694 toxicity (data not shown). However, the TS mismatch siRNA containing dUdU overhangs was unable to reverse drug toxicity. In addition to ZD1694, the cytotoxicity of 5'-fluoro-2'-deoxyuridine was reversed by transfection of the dTdT-containing mismatch but the toxicity of a non-TS-directed inhibitor compound such as cisplatin was not reversed by the mismatch siRNA (data not shown).

**Figure 5:**
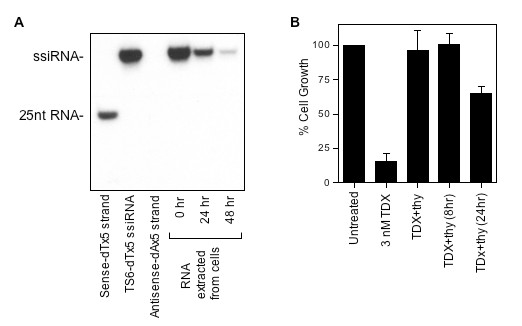
**siRNA northern blot analysis**. **(a) **TS6-dTx5 ssiRNA was transfected into RKO cells. After 6 h, the cell culture medium was replaced. RNA was extracted using Trizol immediately and after an additional 24 and 48 h followed by northern blot analysis as described in the Methods section. **(b) **Thymidine rescue of ZD1694 toxicity. RKO cells were incubated with 3 nM ZD1694 in the presence or absence of 10 uM thymidine. Thymidine (10 μM) was also added 8 and 24 h after ZD1694 addition. After an additional 72 h, cell proliferation was quantified by the WST-1 assay. Cell growth values represent the mean ± standard deviation (SD) from three experiments performed in duplicate.

To determine whether degradation of the ssiRNA was rapid enough to release dTMP for ZD1694 rescue, we performed a series of pulse-chase experiments. RKO cells were transfected with 10 nM TS6-dTx5 ssiRNA. After 6 h, the culture medium was replaced and cellular RNA was extracted after various times followed by northern blot analysis. As seen in Figure 5a, control RNAs (sense and ssiRNA) were readily detected by the antisense probe whereas the antisense RNA was not seen. ssiRNA isolated from transfected RKO cells was also observed at all time points. No smaller RNA degradation products were observed, which may suggest that once the RNA is targeted for degradation, it is rapidly degraded. However, the absence of smaller-sized RNAs may be due to limitations of the siRNA northern blot analysis, as the 25-nucleotide antisense probe may not bind with sufficiently high affinity to smaller sense RNAs. The estimated half-life of the TS6-dTx5 siRNA in RKO cells was 13.2 ± 1.6 h (n = 3). To determine whether this is sufficient time to permit rescue of TS inhibition, cell growth studies were performed with ZD1694 and thymidine. The cytotoxicity of ZD1694 can be completely reversed by thymidine addition 8 h after ZD1694 (Figure 5b). In contrast, the addition of thymidine 24 h after ZD1694 treatment results in only partial rescue. Taken together, these findings suggest that the dT-containing ssiRNA was being rapidly degraded resulting in the intracellular release of dTMP, which can then rescue cells from TS inhibition caused by either siRNA or small molecule inhibitors.

## Discussion

In this study, we investigated the use of 'sticky' siRNAs as potential therapeutic molecules for targeting TS. Previous work by Bolcato-Bellemin *et al*. demonstrated that standard siRNAs are not tightly bound to the cationic delivery molecule PEI and are exchanged for cell surface polyanionic syndecans resulting in their release prior to cellular uptake [13]. They discovered that extension of the 3' end of the sense strand by either five or eight deoxythymidine molecules (with complimentary deoxyadenosine molecules on the antisense strand) allowed these ssiRNAs to be stably complexed into PEI nanoparticles and ultimately released after cellular uptake. Our studies provide further evidence that this modification results in effective delivery of TS6 ssiRNA with linear PEI.

As with any modification to a potential 'drug' molecule, we wanted to ensure that the 3' extensions did not affect the ability of the siRNA to inhibit cell growth. Surprisingly, higher concentrations of TS6 ssiRNA with dT overhangs displayed significantly less inhibitory effect on RKO cell growth. However, transfection of a TS-targeted ssiRNA with dU overhangs maintained the inhibitory effects on cell growth over the concentration range tested. This observation was also true for standard siRNAs with two dTdT nucleotide overhangs. Thus, our findings suggest that as the siRNA is degraded into its nucleotide components, free dTMP is released into cells and eventually metabolized to the dTTP metabolite, which is then able to prevent the cytotoxic effects of the TS-targeted siRNA. This observation provides a reasonable explanation for our earlier work, which showed that cell growth was inhibited by at most 30% using siRNAs containing dTdT overhangs despite a > 95% reduction in TS protein expression [4]. The difference in cell growth observed here compared to this previous work (50% versus 30% growth suppression) may also be attributed to the use of OPTI-MEM medium (Invitrogen). This enhanced culture medium, which is commonly used for *in vitro *transfections, contains 3 μM thymidine, and this level of thymidine may have contributed, in part, to the reduced growth effect of TS6 siRNA.

Given the possibility of cell growth rescue, we determined whether such dTMP release from the degraded siRNA could reverse the cytotoxicity of TS inhibitor compounds. Both siRNAs and ssiRNAs containing dT overhangs reversed ZD1694 toxicity in a dose-dependent fashion. However, the respective siRNA and ssiRNA molecules with dU overhangs were unable to rescue cells providing supportive evidence that the reversal of cell growth inhibition is, in fact, due to dTMP release. We further showed that the ssiRNA is degraded intracellularly with an approximate half-life of 13 h. Exogenous addition of thymidine 8 to 24 h after ZD1694 treatment was able to reverse cytotoxicity, suggesting that the degradation of ssiRNAs provides for sufficiently high levels of free dTMP for drug rescue.

This observation has clear therapeutic relevance as siRNA high-throughput screens are being used to identify possible candidate genes for therapeutic intervention. A screen containing dTdT-siRNAs targeting thousands of genes and utilizing cell proliferation or cell migration as a selection predictor may only identify genes with little to no effect on the TS pathway and/or TS-associated pathways as the dTdTs would reverse growth effects from TS-influenced genes. The use of such screens may therefore result in misinterpretation of the observed findings. Furthermore, this observation has clinical importance as siRNAs are presently being actively developed in clinical trials. A recent clinical study utilized dT-containing siRNAs to prevent lung infection of the respiratory syncytial virus [21]. Given the essential role of TS in cell growth and differentiation, the efficient rescue of its growth effects by dT-siRNAs may have unintended consequences on siRNA therapeutics. Presently, the general consensus is that nearly every gene may be 'targetable' with siRNAs. However, many of these potential therapeutic targets may have either direct or indirect effects on TS expression. For example, inhibition of both epidermal growth factor receptor (EGFR) and HER2 with the small molecule inhibitor lapatanib has been shown to downregulate TS protein expression which, in turn, can then sensitize cells to fluorouracil (5-FU) and the oral fluoropyrimidine capecitabine [22]. There are other potential protein targets that may influence and/or regulate TS expression, and they include calcium-sensing receptor (CaSR), E2F1, C-MYC, late SV40 factor (LSF), astrocyte elevated gene 1 (AEG-1), cyclin-dependent kinase 4 (CDK4), and histone deacetylases (HDAC) [22-29]. With respect to HDACs, clinical trials are ongoing to take advantage of the inhibitory effect of HDAC inhibitors on TS expression by combining them with fluoropyrimidines in various clinical regimens [30]. If a HDAC-specific siRNA was used in place of the small molecule HDAC inhibitor, it is conceivable that the dTdT overhangs would rescue from TS inhibition, resulting in a reduced therapeutic efficacy.

It is certain that as the critical issues of stability and tissue delivery are resolved, siRNAs will move from being a genetic research tool to therapeutic drug molecules. Significant attention has focused on the potential off-target effects of siRNAs. These effects include passenger strand targeting and miRNA-like effects [31]. To our knowledge, this is the first report of off-target effects resulting directly from siRNA degradation. As new nucleotide modifications are incorporated into the siRNA backbone, careful attention must be given as to the potential consequences these degraded modified nucleotides might have on cellular metabolism, signaling, and growth. Based on our findings, siRNAs should be designed so that they incorporate only uridine residues in the 3'-overhang position, especially when the target gene may, in some manner, be related to the TS-signaling pathway.

## Methods

### siRNAs

siRNA and ssiRNA duplexes were obtained from Dharmacon Research (Lafayette, CO, USA). The synthesis of the TS6 siRNA has been previously described [4] (sequences listed in Table 1). The luciferase GL2 siRNA (#D-001100; 5'-CGUACGCGGAAUACUUCGA-'3) and a HER2-targeting siRNA (5'-UCUUAGACGAAGCAUACGU-'3) containing standard dTdT 3'-end overhangs were also obtained from Dharmacon.

**Table 1:** Small interfering RNA (siRNA) sequences

siRNA		Sequence
TS6-dTdT	5'-	GGAUAUUGUCAGUCUUUAGG-dTdT-'3
	3'-dTdT-	CCUAUAACAGUCAGAAAUCC-'5

TS6-dUdU	5'-	GGAUAUUGUCAGUCUUUAGG-dUdU-'3
	3'-dUdU-	CCUAUAACAGUCAGAAAUCC-'5

TS6-dTx5	5'-	GGAUAUUGUCAGUCUUUAGG-dTdTdTdTdT-'3
	3'-dAdAdAdAdA-	CCUAUAACAGUCAGAAAUCC-'5

TS6-dUx5	5'-	GGAUAUUGUCAGUCUUUAGG-dUdUdUdUdU-'3
	3'-dAdAdAdAdA-	CCUAUAACAGUCAGAAAUCC-'5

Mismatch-dTx5	5'-	GGAUA**C**UG**C**CA**A**UCU**C**UAGG-dTdTdTdTdT-'3
	3'-dAdAdAdAdA-	CCUAU**G**AC**G**GU**U**AGA**G**AUCC-'5

These siRNAs target nucleotides 1106-1123 of human thymidylate synthase (TS) mRNA. The top strand is the sense (passenger) strand with the bottom strand being the antisense (guide) strand of the siRNA. Mismatches are shown in bold type.

dA = deoxyadenosine; dT = deoxythymidine; dU = deoxyuridine;.

### Cell culture

The human colon cancer RKO cell line, originally obtained from the American Type Culture Collection (ATCC), has been previously well characterized and was maintained in our laboratory at 37°C in 75 cm^2 ^tissue culture flasks (BD Bioscience, San Jose, CA, USA) in RPMI-1640 growth medium containing 10% dialyzed fetal bovine serum [4]. RKO cells are routinely authenticated by morphology and growth curve analysis. Cells were tested periodically for *Mycoplasma *by the MycoAlert *Mycoplasma *detection assay (Cambrex Bio Science, Rockland, ME, USA).

### siRNA transfection

Cells were plated in six-well plates at a density of 1 × 10^5 ^cells per well. The following day, siRNA duplexes were complexed with LF2000 (Invitrogen, Carlsbad, CA, USA) in serum-free RPMI-1640 medium or linear jetPEI (Polyplus Transfection Inc., New York, NY, USA) in 4-(2-hydroxyethyl)-1-piperazine-ethanesulfonic acid (HEPES)-buffered saline as described by the manufacturer's protocol and added to the plated cells. After 48 h, the wells were rinsed with phosphate-buffered saline (PBS), and cells were scraped in cell lysis buffer (10 mM Tris pH 7.4, 150 mM NaCl, 1 mM ethylenediaminetetra-acetic acid (EDTA), 1% octylphenoxypolyethoxyethanol (IGEPAL), 0.5% deoxycholic acid, and 0.1% SDS) containing freshly added Protease Inhibitor Cocktail (Sigma, St Louis, MO, USA) and 1 mM phenylmethanesulfonylfluoride (PMSF). Lysates were sonicated three times at 3 s each and centrifuged at 10,000 *g *for 10 min at 4°C. Cell lysates were stored at -80°C for future use. For mRNA analysis, cells were rinsed with PBS, and incubated with Trizol (Invitrogen). Total RNA was stored at -80°C for future use.

### Western immunoblot analysis

Protein concentrations of cell lysates were determined using the DC Protein Assay (Bio-Rad, Hercules, CA, USA). Equivalent amounts of protein (50 μg) from each cell lysate were resolved on SDS-PAGE using the method of Laemmli [32]. Gels were electroblotted onto nitrocellulose membranes (0.45 μm; Bio-Rad), and membranes were then incubated in blocking solution (1XPBS, 0.1% Tween-20, 5% non-fat dry milk powder) for 1 h at room temperature. Membranes were incubated at 4°C overnight with primary antibodies at the following dilutions: anti-TS monoclonal antibody, 1:5,000 (Zymed Laboratories, San Francisco, CA, USA); anti-α-tubulin monoclonal antibody 1:5,000 (EMD Biosciences, Gibbstown, NJ, USA). After multiple 1XPBS, 0.1% Tween-20 (PBST) washes, membranes were incubated with a dilution of 1:10,000 of horseradish peroxidase-conjugated secondary antibody (goat anti-mouse IgG; Bio-Rad) for 1 h at room temperature. After additional PBST washes, membranes were processed by the enhanced chemiluminescence method (SuperSignal West Pico substrate; Pierce, Rockford, IL, USA), and protein bands were visualized by autoradiography. Quantitation of signal intensities was performed by densitometry on a Hewlett Packard ScanJet 5370C (Hewlett Packard, Palo Alto, CA, USA) using NIH ImageJ software (http://rsbweb.nih.gov/ij/).

### Cell growth assays

RKO cells were plated in 96-well plates at a density of 800 cells/well. The following day, cells were transfected with siRNA/LF2000 complexes and allowed to incubate for 96 h. Cell proliferation and viability was quantified by the WST-1 assay (Roche Applied Science, Indianapolis, IN, USA). For ZD1694 rescue experiments, a concentration of 3 nM ZD1694 was added simultaneously with the siRNA complexes.

### Real-time quantitative reverse transcriptase (qRT)-PCR analysis

The first-strand cDNA was synthesized using 0.5 μg total RNA and the Quantitect Reverse Transcription Kit (Qiagen Inc., Valencia, CA, USA). The real-time PCR was performed in triplicates using the Taqman Gene Expression Master Mix (Applied Biosystems, Foster City, CA, USA) in a final reaction volume of 20 μl with gene-specific primer/probe sets, and a standard thermal cycling procedure (40 cycles) on a Mastercycler EP realplex instrument (Eppendorf, Hamburg, Germany). The mRNA level of TS and 18s RNA was assessed using the TaqMan Gene Expression real-time PCR assays (Applied Biosystems, assay IDs: Hs00426586_m1 and Hs03928990_g1, respectively). The results were expressed as the threshold cycle (CT). The relative quantification of the target transcripts was determined by the comparative Ct method (∆∆Ct) according to the manufacturer's protocol (User Bulletin No. 2, Applied Biosystems). The 2^-^^∆^^∆^^Ct ^method was used to analyze the relative changes in gene expression between LF2000-treated cells and siRNA/LF2000-treated cells. Control experiments without reverse transcription were performed to confirm that the total RNA contained no genomic DNA contamination.

### siRNA northern blot analysis

RKO cells were transfected with 10 nM TS6-dTx5 ssiRNA complexed with LF2000. After 6 h, the transfection medium was removed and total RNA was extracted from cells using Trizol. For 24 and 48 h time points, fresh growth medium was added back to the wells, and cells were harvested for total RNA at time. Total RNA (2-5 μg) was loaded onto a native 20% acrylamide gel and electrophoresed for 90 min at 450 V. Single-stranded and double-stranded RNAs were included in each gel as negative and positive controls. To account for RNA dilution due to cell growth, total RNA isolated from the same number of cells was used for each time point. The RNAs were transferred onto BrightStar-Plus positively charged nylon membrane (Ambion, Austin, TX, USA) using the GENIE blotter (Idea Scientific, Minneapolis, MN, USA) in 1XTris/borate/EDTA (TBE). The antisense strand of TS6 ssiRNA was 5'-end labeled using T4 kinase (Promega, Madison, WI, USA) and [P^32^]-ATP. After prehybridizing the membrane, the antisense probe was added to Northern Max Hybridization Buffer (Ambion) and incubated with the membrane at 37°C overnight. The membrane was washed with 2X saline-sodium citrate (SSC)/0.1% SDS and 0.2X SSC/0.1% SDS buffer before being exposed to film. Quantitation of signal intensities was performed by densitometry on a Hewlett Packard ScanJet 5370C using NIH ImageJ software.

## Competing interests

The authors declare that they have no competing interests.

## Authors' contributions

JCS conceived the study, performed the experiments, and drafted the manuscript. EC participated in the study design, drafted, and revised the manuscript for intellectual content. Both authors read and approved the final manuscript.
